# Binding properties of *Treponema denticola* lipooligosaccharide

**DOI:** 10.3402/jom.v5i0.21517

**Published:** 2013-09-16

**Authors:** Daniel Grenier

**Affiliations:** Groupe de Recherche en Écologie Buccale, Faculté de Médecine Dentaire, Université Laval, Quebec, Canada

**Keywords:** adherence, cytokine, epithelial cells, fibroblasts, laminin, lipooligosaccharide, periodontal disease, Peptostreptococcus micros, Treponema denticola

## Abstract

**Background and objective:**

The cell-surface lipooligosaccharide (LOS) of *Treponema denticola* possesses several biological properties. The aim of this study was to investigate the binding properties of *T. denticola* LOS to extracellular matrix (ECM) proteins, mucosal cells, and oral bacteria.

**Design:**

LOS was isolated from *T. denticola* and labeled with tritium. Tritium-labeled LOS was placed in ECM protein-, epithelial cell-, fibroblast-, or bacterium-coated wells of a 96-well microplate. Following incubation, unattached LOS was removed by extensive washing, and the amount of bound LOS was determined by measuring the radioactivity in the wells. *Peptostreptococcus micros* coated with LOS was used to stimulate fibroblasts, and the secretion of interleukin-6 (IL-6) and interleukin-8 (IL-8) by the fibroblasts was determined by ELISA.

**Results:**

*T. denticola* LOS had a high affinity for laminin. It also bound to gingival epithelial cells and fibroblasts. Soluble CD14 significantly increased the binding of LOS to fibroblasts. More LOS bound to *P. micros* than the other oral bacterial species tested. Stimulating fibroblasts with LOS-coated *P. micros* induced the secretion of IL-6 and IL-8.

**Conclusions:**

Our study provided evidence that *T. denticola* LOS possesses the capacity to bind to ECM proteins, mucosal cells, and oral bacteria. In addition, LOS binding to bacteria may increase their pro-inflammatory potential.

Chronic periodontitis is a form of inflammatory periodontal disease that affects a large proportion of the population and causes the destruction of tooth-supporting tissues, including the periodontal ligament and alveolar bone. *Treponema denticola*, which is often found in association with *Porphyromonas gingivalis* and *Tannerella forsythia*, is a major etiological agent of chronic periodontitis (1, 2). *T. denticola* produces several virulence factors that can promote host colonization and invasion, perturb the host defense system, and destroy periodontal tissues (3, 4).

The cell-surface lipooligosaccharide (LOS) of *T. denticola* has chemical properties that differ from those reported for classic Gram-negative lipopolysaccharide (LPS) (5). *T. denticola* LOS has a diacylglycerol lipid anchor and a hexose–hexosamine–hexose core region, but lacks heptose, 3-deoxy-d-manno-2-octulosonic acid, and β-hydroxy fatty acids, which are core components of LPS (5). *T. denticola* LOS has been reported to activate various mammalian cell types. It stimulates osteoclastogenesis and matrix metalloproteinase (MMP) expression in a mouse calvaria/bone marrow cell co-culture model (6) and induces inflammatory mediator production by murine macrophages (7). It was recently shown that *T. denticola* LOS induces the secretion of several inflammatory mediators as well as MMP-3 by gingival fibroblasts through the activation of specific signaling pathways (8).

The ability of *T. denticola* to efficiently colonize and establish itself in subgingival sites is dependent on its capacity to adhere to mucosal cells and extracellular matrix (ECM) proteins (3, 4, 9). Two well-characterized outer membrane components of *T. denticola* involved in adherence are the major surface protein (Msp) and the oligopeptide-binding protein homologue (OppA) (9). Both Msp (10) and OppA (11) can bind to host tissue proteins, including fibronectin and laminin, which in turn may enable *T. denticola* to adhere to mucosal cells and the ECM in periodontal sites. In this study, we hypothesized that *T. denticola* LOS possesses binding properties that may contribute to the adherence of this spirochete.

## Materials and methods

### Isolation of LOS

*T. denticola* ATCC 35405 was grown in new oral spirochete (NOS) medium (12) at 37°C for 4 days under anaerobic conditions (80% N_2_, 10% H_2_, 10% CO_2_). LOS was isolated using the protocol of Darveau and Hancock (13), which was initially developed for the isolation of bacterial LPS. The LOS preparation was freeze dried and stored at −20°C. Contaminating protein in the LOS preparation was evaluated at less than 0.001% (w/w) using a DC protein assay kit (Bio-Rad Laboratories, Mississauga, Ontario, Canada). Analysis of the LOS preparation by sodium dodecyl sulphate–12.5% polyacrylamide gel electrophoresis revealed the characteristic profile previously reported (8).

### Preparation of tritium-labeled LOS

*T. denticola* LOS was labeled using a slight modification of the protocol described by Rokita and Menzel (14). Briefly, LOS (1 mg/ml) was dissolved in 100 mM sodium carbonate buffer (pH 9.0) containing 100 mM NaCl. The mixture was vigorously vortexed (5 min) to solubilize the LOS. ^3^H-acetic anhydride in toluene (100 mCi/ml; 500 mCi/mmol; Amersham Pharmacia Biotech Canada, Baie d'Urfé, QC, Canada) was diluted to a final concentration of 1.25 µCi/ml (2.4 µM). After a 1-h incubation at room temperature with gentle shaking, the LOS solution was diluted 1:4 in 100 mM phosphate-buffered saline (PBS, pH 7.2) and was dialyzed (6–8 kDa molecular mass cutoff) seven times at 4°C for 16 h against 4 l of PBS until no significant radioactivity was detected in the dialysate using a multi-purpose scintillation counter (Beckman Coulter, Fullerton, CA). The specific activity of the tritium-labeled *T. denticola* LOS preparation was 47 µCi/mg of LOS, which corresponds to 92,500 dpm/µg. The preparation was aliquoted and stored at −20°C.

### Binding to immobilized human tissue proteins

Stock solutions (100 µg/ml) of fibronectin and laminin were prepared in 50 mM Tris-HCl buffer (pH 7.4) while those of type I collagen and type IV collagen were prepared in 0.01% acetic acid. Bovine serum albumin was used as a negative control. All proteins were purchased from Sigma-Aldrich Canada Co. (Oakville, Ontario, Canada). The protein solutions (100 µl) were added to the wells of a flat-bottomed 96-well microplate (MaxiSorp BreakApart Module, Nalge Nunc International Corp., Rochester, NY), and the plate was incubated for 2 h at 37°C. The protein solutions were removed by aspiration, and the wells were washed three times (5 min with gentle shaking) with PBS. Tritium-labeled LOS (5 µg in 50 µl) was added to each well, and the microplate was incubated for 90 min at 37°C. The wells were then washed five times with 100 µl of PBS to remove unbound LOS. Individual wells were snapped off the plate and were placed in vials containing scintillation liquid to determine the amount of radioactivity corresponding to bound LOS. The background value of radioactivity obtained with bovine serum albumin (negative control) was substracted. All assays were performed in triplicate, and the mean±standard deviations (SD) were calculated.

### Binding to human gingival epithelial cells and fibroblasts

The immortalized human oral epithelial cell line GMSM-K was kindly provided by Dr. Valerie Murrah (University of North Carolina, Chapel Hill, NC). This cell line has an epithelial phenotype based on electron microscopic and immunohistochemical analyses (15). Primary human gingival fibroblasts HGF-1 were purchased from the American Type Culture Collection (ATCC, Manassas, VA). Both cell lines were cultured in Dulbecco's modified Eagle's medium (DMEM) supplemented with 4 mM l-glutamine (HyClone Laboratories, Logan, UT), 10% heat-inactivated fetal bovine serum (FBS, Sigma-Aldrich), and 100 µg/ml of penicillin G-streptomycin, and were incubated at 37°C in a 5% CO_2_ atmosphere. Epithelial cells were seeded at a concentration of 4×10^5^ cells/ml and fibroblasts at a concentration of 1×10^5^ cells/ml in the wells of flat-bottomed 96-well microplates. Confluent epithelial cells and fibroblasts were incubated with tritium-labeled LOS (4 µg in 50 µl) for 90 min at 37°C in a 5% CO_2_ atmosphere. In one assay, soluble CD14 (10, 20, or 40 ng/ml) (Feldan, Quebec, Canada) was also added to the wells. The wells were then washed five times with 100 µl of PBS to remove unbound LOS. Radioactivity was determined as described above. The background value of radioactivity obtained with bovine serum albumin-coated wells (negative control) was substracted. All assays were performed in triplicate, and the mean±SD were calculated.

### Binding to immobilized bacteria

*Actinomyces viscosus* ATCC 27044, *Peptostreptococcus micros* 1251 and 1262, *P. gingivalis* ATCC 33277, *Streptococcus mitis* ATCC 33399, *Streptococcus mutans* ATCC 25175, *Streptococcus salivarius* ATCC 25975, and *Streptococcus sanguinis* NY101 were grown in Todd–Hewitt broth (BBL Microbiology Systems, Cockeysville, MD) for 24 h and were suspended in 25 mM carbonate buffer (pH 9.6) to an optical density at 660 nm of 0.5. The wells of a flat-bottomed 96-well microplate were filled with the bacterial suspensions (100 µl), and the plate was incubated for 2 h at 37°C. The bacterial suspensions were removed by aspiration, and the wells were washed three times (5 min with gentle shaking) with PBS. Bacteria that had adhered to the wells were then fixed with 0.05% glutaraldehyde for 30 min at room temperature. The wells were washed three times with PBS, tritium-labeled LOS was added, and bound radioactivity was measured as described above. The background value of radioactivity obtained with bovine serum albumin-coated wells (negative control) was substracted. All the assays were performed in triplicate, and the mean±SD were calculated.

### Stimulation of gingival fibroblasts with LOS-coated *P. micros*

Human gingival fibroblasts (HGF-1) were grown to confluence as described above. Equal volumes (500 µl) of a *P. micros* 1262 suspension in DMEM-1% FBS (OD_660_=0.25) and unlabeled *T. denticola* LOS (10 µg/ml in PBS) were mixed and were incubated at 37^o^C for 1.5 h. Unbound and loosely attached LOS was removed by three consecutive centrifugations (10,000 g for 10 min). The *P. micros* cells were then suspended in the original volume of DMEM-1% FBS. The fibroblast medium was aspirated and was replaced by the *P. micros* suspension. After a 24-h incubation (37°C, 5% CO_2_), the culture medium supernatants were collected and were stored at −20°C until used. Fibroblasts stimulated in the presence of polymyxin B (10 µg/ml) were used as controls. Commercial enzyme-linked immunosorbent assay (ELISA) kits (R&D Systems, Minneapolis, MN) were used to quantify IL-6 and IL-8 concentrations in the cell-free culture supernatants according to the manufacturer's protocols. The sensitivities of the commercial ELISA kits were 9.3 pg/ml for IL-6 and 31.2 pg/ml for IL-8. Cytokine concentrations were determined in triplicate, and the mean±SD were calculated.

### Statistical analysis

Differences between mean±SD were analyzed for statistical significance using the Student's *t*-test.

## Results and discussion

Given the specific activity of the tritium-labeled *T. denticola* LOS preparation, a standard curve was generated and used to estimate bound LOS in the binding assays. We first tested the binding of *T. denticola* LOS to various ECM proteins. As shown in Table 1, LOS had a high affinity for laminin, with 357±22 ng of LOS binding to laminin-coated wells. *T. denticola* LOS also bound to fibronectin and type I collagen, albeit to a much lower extent, but did not bind to type IV collagen. Previous studies have reported that *T. denticola* cells bind to laminin (16, 17), which may be one mechanism by which this spirochete adheres to host cells and the ECM. Our study suggests that, like Msp (10) and OppA (11), LOS may play a role in *T. denticola* binding to laminin.

**Table 1:** Binding of tritium-labeled *T. denticola* LOS to human ECM proteins immobilized in the wells of a microplate

Protein	Bound *T. denticola* LOS (ng)
Type I collagen	17±9
Type IV collagen	ND
Fibronectin	37±12
Laminin	357±22

ND, not detected.

Bacterial adherence to host cells is a critical step in the pathogenesis of periodontitis. It is also the initial step for the internalization process associated with host cell invasion. In our study, similar amounts of LOS bound to monolayers of gingival epithelial cells (137±14 ng) and fibroblasts (159±24 ng) (Table 2). It has previously been reported that *T. denticola* adheres to human gingival fibroblasts (18) and epithelial cells (19). While we provided evidence that LOS may be involved in the adherence of *T. denticola* to epithelial cells, it is likely that other cell-surface components expressed by this spirochete contribute to adherence. In agreement with our results, LOS also plays a role in the adherence of *Moraxella catarrhalis* (20) and *Haemophilus parainfluenzae* (21) to epithelial cells. Interestingly, the ability to adhere to and invade human conjunctival cells is significantly impaired in an LOS-deficient mutant of *M. catarrhalis* (20). In addition, the LOS of *Haemophilus ducreyi* is involved in the adherence of this bacterial species to foreskin fibroblasts (22).

**Table 2:** Binding of tritium-labeled *T. denticola* LOS to gingival epithelial cells and fibroblasts in the presence and absence of soluble CD14

	Bound *T. denticola* LOS (ng)	
	
Amount of soluble CD14 (ng/ml)	Epithelial cells	Fibroblasts
0	137±14	159±24
10	141±32	212±84
20	139±28	359±62*
40	203±71	422±49*

* Significant increase (*P*<0.01) compared to the control without soluble CD14.

CD14, a membrane-anchored glycoprotein expressed by various cell types, is a high-affinity receptor for bacterial LPS (23). Given that higher concentrations of soluble CD14 have been found in gingival crevicular fluid (24, 25), we tested the impact of exogenous soluble CD14 on the binding of *T. denticola* LOS to epithelial cells and fibroblasts. While there was no significant effect on binding to epithelial cells, soluble CD14 dose dependently increased the binding of LOS to fibroblasts (Table 2). For example, the highest concentration of soluble CD14 tested (40 ng/ml) increased LOS binding to fibroblasts by 2.7-fold.

Finally, we investigated the binding of *T. denticola* LOS to bacteria immobilized in the wells of a microplate. As shown in Table 3, *T. denticola* LOS had the most affinity for *P. micros*, with 404±31 ng binding to *P. micros* 1262 and 452±37 ng to *P. micros* 1251. It also bound to some extent to *S. salivarius, S. mutans, S. mitis*, and *A. viscosus*.

**Table 3:** Binding of tritium-labeled *T. denticola* LOS to bacteria immobilized in the wells of a microplate

Bacteria	Bound *T. denticola* LOS (ng)
*A. viscosus* ATCC 27044	31±12
*P. micros* 1251	452±37
*P. micros* 1262	404±31
*P. gingivalis* ATCC 33277	ND
*S. mitis* ATCC 33399	33±9
*S. mutans* ATCC 25175	54±15
*S. salivarius* ATCC 25975	94±29
*S. sanguinis* NY101	ND

ND, not detected.

The secretion of IL-6 and IL-8 by gingival fibroblasts stimulated with *P. micros* treated or not with *T. denticola* LOS is reported in Table 4. When stimulated with *P. micros* alone, 91±32 pg/ml and 165±26 pg/ml of IL-6 and IL-8 were secreted, respectively. Pretreating *P. micros* with *T. denticola* LOS resulted in a 4.8-fold increase in IL-6 secretion and a 3.6-fold increase in IL-8 secretion relative to untreated *P. micros* cells. This effect was suppressed by the presence of polymyxin B. Yoshioka et al. (26) have reported that *Aggregatibacter actinomycetemcomitans* LPS binds to the surface of *P. micros* cells and that LPS-coated *P. micros* can induce tumor necrosis factor alpha (TNF-α) secretion by human U937 monocytic cells differentiated into adherent macrophages (26).

**Table 4:** Cytokine secretion by human gingival fibroblasts stimulated with *T. denticola* LOS-coated *P. micros* cells.

	Amount of cytokine secreted (pg/ml)	
	
Stimulation condition	IL-6	IL-8
Control	ND	31±9
*P. micros*	91±32	165±26
*T. denticola* LOS-coated *P. micros*	437±75*	594±91*
*T. denticola* LOS-coated *P. micros+*polymyxin B	123±42	245±84

ND, not detected.

* Significant increase (*P*<0.01) compared to the stimulation with *P. micros* alone.

In conclusion, our study provided evidence that *T. denticola* LOS possesses the capacity to bind to ECM proteins, mucosal cells, and oral bacteria. This may represent a new mechanism by which this spirochete may colonize periodontal sites. In addition, LOS binding to bacteria may increase their pro-inflammatory potential.
